# Sphincter repair procedures may be favored in the treatment of obstetrical recto-vaginal fistula: a systematic review of the literature and meta-analysis

**DOI:** 10.1007/s10151-025-03133-3

**Published:** 2025-04-07

**Authors:** A. Venara, E. Houlet, E. Poupard, M. André, P. E. Bouet, J. Gillet, J. F. Hamel

**Affiliations:** 1 Faculty of Health, Department of Medicine, University of Angers, Angers, France; 2 Department of Digestive Surgery, University Hospital of Angers, 4 rue Larrey, Angers Cedex 9, 49933 Angers, France; 3 IHFIH, UPRES, University of Angers, 3859 Angers, EA France; 4 The Enteric Nervous System in Gut and Brain Disorders, Université de Nantes, INSERM, TENS, IMAD, 44000 Nantes, France; 5 Department of Gynecology and Obstetrics, University Hospital of Angers, 4 rue Larrey, angers cedex 9, 49933 Angers, France; 6 Department of Biostatistics, La Maison de La Recherche. University Hospital of Angers, 4 rue Larrey, Angers Cedex 9, 49933 Angers, France; 7 Department of Visceral Surgery, CHU Angers, 4 rue Larrey, 49933 Angers Cedex 09, 49933 Angers, France

**Keywords:** Obstetric, Anovaginal fistula, Rectovaginal fistula, Repair

## Abstract

**Background:**

The management of obstetric rectovaginal fistula (RVF) is challenging for the surgeon. The best surgical procedure to repair RVFs, specifically after obstetric anal sphincter injury, has not been extensively studied. The objective was to compare the success of the different procedures performed to repair obstetric RVF.

**Methods:**

The literature search was carried out on PubMed^®^ and Web of Science^®^ from database inception until 31 December 2022. Selection criteria were: (1) patients with a diagnosis of obstetric-related RVF; (2) patients treated surgically with no restriction concerning the considered surgery; (3) clinical trials or epidemiological studies. Meta-analysis was conducted considering the network meta-analysis framework to allow studying the relative value of each treatment mentioned in the selected articles.

**Results:**

The quantitative synthesis included 32 studies (18 retrospective and 14 prospective) accounting for 595 patients. The quality of these studies was low because of the lack of prospective randomization. Nineteen procedure types were described and assessed. Most patients (*n* = 180) underwent endorectal advancement flap (ERAF) followed by excision and layered closure (ELC) (*n* = 213) and Musset procedure (*n* = 65). A diverting stoma was performed in 66/132 patients. Only 13 studies reported the functional results of the procedure. In the meta-analysis, the Musset procedure (OR = 4.29; 95% CI: 1.18–16.14), transvaginal ELC (OR = 11.84; 95% CI: 2.18–91.80) and transperineal ELC (OR = 3.56; 95% CI: 1.26–10) significantly improved the anatomical results compared to ERAF.

**Conclusions:**

A further randomized controlled trial in the literature assessing ERAF and sphincteroplasty to compare the anatomical results, functional results and morbidity of this treatment is needed.

**Registration:**

PROSPERO CRD42023447875.

**Supplementary Information:**

The online version contains supplementary material available at 10.1007/s10151-025-03133-3.

## Introduction

Obstetrical genito-urinary and recto-vaginal fistulas (RVFs) are reported to occur in 0 to 4.09 per 1000 births in low- and middle-income countries (LMICs) [1, 2]. In Western countries, RVF complicates 1–3% [3] of anal sphincter injuries (OASI) occurring in 0.5–4.5% of deliveries [4]. The mechanism of RVF may be different in LMICs and Western countries. Indeed, in Western countries, RVF is secondary to OASI or wound dehiscence, and it is carried out because of prolonged labor leading to necrosis of the perineum secondary to crushing by the fetal head in LMICs.

As obstetric RVF is the main cause of RVF, specific guidelines to treat this complication would be expected. However, the management of this RVF is challenging for the surgeon as no guidelines have been published. Current literature lacks studies focusing exclusively on the obstetric etiology of RVFs, while Karp et al. [5] report that non-obstetric RVFs have a nearly fourfold increased risk of repair failure compared with obstetric fistulas. The results of studies not focusing on obstetric causes should therefore be interpreted with caution as the other causes of RVF may impair outcomes of the proper surgical procedure.

In a recent nationwide retrospective cohort, Venara et al. reported that more than ten procedures can be performed to cure RVFs, from low-invasive sphincter-sparing procedures to gracilis flaps and the Musset procedure [6]. In this report, the most performed procedure was the sphincter-sparing endorectal advancement flap (ERAF) procedure [6] in accordance with the American Society of Colon and Rectal Surgeons (ASCRS) recommendations for the treatment of anoperineal fistulas [7]. However, this latter procedure is successful in only 22–50% [6, 8, 9] of cases, which calls for new guidelines for this specific disease.

To develop guidelines, the strongest evidence is needed, and a review of the literature with a meta-analysis may facilitate this. The main aim of this systematic review of the literature and meta-analysis was to compare the success of the different procedures performed to repair obstetric RVF. The secondary aim was to give an overview of the role of the diverting stoma in RVF healing.

## Methods

### Search strategy

The present review and meta-analysis were conducted according to the recommendations of the PRISMA statement for reporting systematic reviews and meta-analyses of studies [10]. It was registered with PROSPERO.

Systematic searches were performed in 2023 in two different databases: PubMed^®^ and Web of Science^®^. Studies published from database inception to 31 December 2022 in English or French language were considered. A combination of MeSH terms and text words was used: ((rectovaginal) OR (anovaginal) OR (recto-vaginal) OR (ano-vaginal)) AND (fistula) AND (obstetric*). Reference lists of included articles were considered to identify any other trials. Experts were consulted to ensure that all key studies were included.

Studies that met the following criteria were included in the systematic review: (1) patients with a diagnosis of obstetric-related RVF, (2) patients treated surgically with no restrictions concerning the considered surgery and (3) clinical trials or epidemiological studies (prospective observational, cohort, cross-sectional or retrospective).

Studies were excluded if (1) they were case reports, (2) they were not a full-text paper (i.e., exclusion of reviews, protocol publications, abstracts, letters and comments), (3) the study did not report results specifically for the specific obstetric-related subsample for studies focusing on a population of patients including both obstetric-related and non-obstetric-related rectovaginal fistula, (4) the surgical procedure was not described and (5) the study involved animals. No other restriction was used. When the studies were not available, the authors were contacted to request the manuscript.

### Data extraction

All results were screened for duplication by author, title, journal and publication date. Selected articles were screened for relevance in a hierarchical procedure first by title, then abstract and finally by the full text of all studies previously considered relevant. This procedure was conducted independently by two different co-author groups: EP and AM; EH and JG. In case of any discrepancy, a third author (AV) reviewed the article for final assessment.

Data were extracted from the included studies to a standard sheet including the first author, year of publication, study design, number of patients enrolled, number of patients enrolled with a diagnosis of obstetric RVF, success rate of the surgery and type of procedure, functional success of the procedure, construction of a diverting stoma and Joanna Briggs Institute (JBI) critical appraisal tool components. Two co-authors independently collected these data (EH and JG).

The main outcome measure was the success of the surgical procedure defined by the anatomical correction of the defect and the absence of stool and/or gas passing in the vagina. The secondary outcomes were: (1) the functional success of the procedure defined by the absence of postoperative fecal incontinence and (2) the construction of a diverting stoma.

### Quality assessment

The quality assessment was performed according to the JBI critical appraisal tool for cohort and case series [11].

### Data synthesis and analysis

Only papers mentioning the results of the surgery in terms of anatomical correction of the defect, absence of postoperative fecal incontinence and/or of construction of a diverting stoma were selected for the meta-analysis. Meta-analysis was conducted considering the network meta-analysis framework to facilitate the study of the relative value of each of the treatments mentioned in the selected articles. Arm-based network meta-analyses based on the Zhang et al. Bayesian hierarchical models, suitable for binary outcomes, were used for performing both direct and indirect comparisons of treatment effects [12]. Results were presented as absolute percentage of success (depending on the considered endpoint) and odds ratios considering the most commonly used surgery for obstetric-related RVF treatment (i.e., the ERAF procedure) as the reference treatment.

## Results

### Study selection

**Fig. 1:**
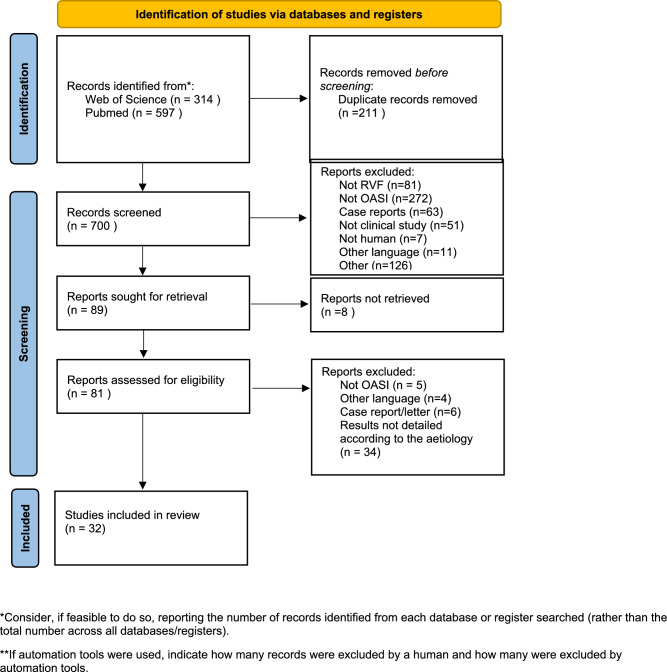
PRISMA flow chart

The flow chart in Fig. 1 depicts the study selection process. A total of 911 studies were identified through PubMed^®^ and World of Science^®^, of which 211 were removed because of duplication. Another 600 articles were excluded because they did not meet the inclusion criteria. The other reasons reported in the flow chart were used to group the studies for which no abstract was available and those that did not describe the treatment, the procedure used for surgery or the etiology of RVF.

After screening the studies according to the abstracts and titles, 89 were selected, of which 8 were excluded because the manuscript was not available. Finally, 32 studies were included in the review and meta-analysis.

### Systematic review

Among the 32 studies included, 18 were retrospective studies [5, 6, 8, 9, 13–25] and 14 were observational prospective studies [26–40]. The design was multicentric in four studies. The main results of the different studies included are reported in Tables 1 and 2, respectively, for the retrospective and prospective studies.

**Table 1:** Characteristics of the different retrospective articles included in the literature review

Articles	Number of patients	Patient age	Setting of the study	Follow-up
Venara et al. [6]	n = 60 patients/*n* = 148 procedures	Mean = 36.2 ± 10.6 years	Multicentric	12 months (± 18) from the date of the last surgery
Beksac et al. [13]	n = 8/19	Mean = 27.63 ± 7.17 years	Monocentric	ND
Reisenauer et al. [14]	n = 13/48	Between 21 and 39 years	Monocentric	Between 1 and 10 years, depending on the time when the fistula repair was done
Hull et al. [9]	n = 54/87	ND	Monocentric	Mean: 49.2 ± 39.2 months
Songne et al. 2007 [15]	n = 1/14	ND	Monocentric	Mean: 40 months (range: 8–120 months)
Devesa et al. [16]	n = 4/46	ND	Monocentric	Median: 8.6 years (range, 2–22 years)
Chew et al. [17]	n = 7/7	Mean: 34 years (range: 22–72)	Bicentric	Mean: 24 months (range: 11–35)
Rhaman et al. [18]	n = 52/52	Mean: 29.5 years (range 17–65)	Monocentric	Median: 24 months min–max: 6 months to 8 years)
Sonoda al. [8]	n = 5/37	ND	Monocentric	Median: 17.1 months (range: 0.4–66.9)
Soriano et al. [19]	n = 25/48	ND	Monocentric	Between 1 and 3 years
Ozuner et al. [20]	n = 13/52	ND	Monocentric	Median: 31 months (± 21.4)
MacRae et al. [21]	n = 14/28	ND	Monocentric	Mean: 11.8 months (range, 2–53)
Reisenauer et al. [22]	n = 29/29	Mean: 29.27 years (range: 21–38)	Monocentric	Up to 14 years
Karp al. [5]	n = 53/88	Median: 37 years (IQR:32.0–47.0)	Monocentric	Median: 157.0 days (range, 47.5–402.0)
Zmora et al. [23]	n = 1/5	35 years	Monocentric	9 months after the surgery and 1 month after the stoma closure
FrontalI et al. [25]	n = 10/51	Mean: 42 years (± 12)	Bicentric	Mean: 56 ± 48 months (range 1–183)
Corman et al. [24]	n = 28/28	Mean 36 years (range:20–66)	Monocentric	3 months to 3 years

Articles	Procedures performed (*N* performed/*N*)	Anatomical success	Functional results (no anal incontinence)	Diverting stoma	Morbidity
Venara et al. [6]	ERAF: n = 49/148, gracilis interposition n = 3/148 Insertion of glue n = 10/148 Ovesco clip n = 9/148 Vaginal suture or flap n = 8/148 Plug n = 7/148 Seton ablation n = 14/148 Musset procedure n = 23/148 Martuis flap n = 16/148	*n* = 11/49 n = 2/3 n = 4/10 n = 3/9 n = 3/8 *n* = 3/7 n = 4/14 n = 15/23 n = 8/16	ND	*n* = 29/60 (26 closures)	ND
Beksac et al. [13]	Transperineal ELC + sphincteroplasty: n = 8/8	*n* = 8/8	*n* = 8/8	None	None
Reisenauer et al. [14]	Transperineal ELC n = 3/13 Transvaginal ELC n = 7/13 Martius flap n = 1/7 Suture of the fourth degree perineal injury n = 1/13	*n* = 3/3 n = 7/7 n = 1/1 n = 1/1	ND	*n* = 4/13 (4 closures)	ND
Hull et al. [9]	ERAF n = 18/54 Transperineal ELC n = 36/54	*n* = 10/18 n = 29/36	ND	ND	*n* = 11/47 (all etiologies)
Songne et al. 2007 [15]	Martius flap n = 1	*n* = 1/1	ND	*n* = 1/1	*n* = 3/14 (all etiologies)
Devesa et al. [16]	ERAF n = 2/4 Transperineal ELC n = 1/4 Rectal and vaginal flap; sphincteroplasty and colostomy; colostomy take-down n = 1/4	*n* = 2/2 n = 1/1 n = 1/1	ND	*n* = 1/4	None
Chew et al. [17]	Transperineal ELC + overlapping sphincteroplasty n = 7/7	*n* = 5/6	*n* = 4/5	None	ND
Rhaman et al. [18]	Transvaginal ELC n = 39/52 - Musset procedure n = 8/52 - Healed spontaneously n = 5/52	*n* = 39/39 n = 8/8 n = 5/5	*n* = 44/52	None	Urinary infection *n* = 4 urinary retention *n* = 2 wound infection *n* = 3 anal incontinence *n* = 2
Sonoda al. [8]	ERAF 5/5	*n* = 2/5	ND	None	ND
Soriano et al. [19]	Musset procedure n = 25/25	*n* = 24/25 (after 2 pocedures for 2 patients)	ND	ND	*n* = 2/48 (all etiologies)
Ozuner et al. [20]	ERAF n = 13/13	*n* = 10/13	ND	ND	ND
MacRae et al. [21]	ERAF n = 9/14 ( - transperineal ELC + sphincteroplasty: n = 5/13 - Coloanal anastomosis n = 2/14	*n* = 4/9 n = 5/5 n = 2/2	ND	ND	ND
Reisenauer et al. [22]	Transvaginal ELC n = 12/29 + sphincteroplasty n = 3/29 - LIFT n = 1/29 - Musset procedure (with levatorplasty) n = 9/29 - Transvaginal ELC + Martius flap interposition n = 2/29 - spontaneous recovery n = 2/29	*n* = 10/12 n = 3/3 n = 1/1 n = 8/9 n = 2/2	ND	*n* = 10/29 (9 closures)	ND
Karp al. [5]	Transperineal ELC n = 53/53 (22/53 + sphincteroplasty)	n = 47/53	ND	None	n = 3/6 (wound infections)
Zmora et al. [23]	Gracilis flap: n = 1	n = 1/1	ND	n = 1/1 (1 closure)	None
FrontalI et al. [25]	Gracilis flap n = 10/10	n = 7/10	ND	n = 10/10	ND
Corman et al. [24]	Transperineal ELC n = 28/28 (levator plication)	n = 28/28	*n* = 28/28	None	Small wound sinus under the skin flap (n = 1)

*ERAF* endorectal advancement flap, *ELC* excision and layered closure, *IQR* interquartile range

**Table 2:** Characteristics of the different prospective articles included in the literature review

Articles	Number of patients	Patients' age	Setting of the study	Follow-up
Norderval et al. [26]	*n* = 9/27	ND	Bicentric	Median: 20 months (inter quartile range (IQR) 14–38 mois)
Mukwege et al. [28]	*n* = 8/10	ND	Monocentric	Mean: 14.3 months (11–36) (all the etiologies)
De Weerd et al. [29]	*n* = 4/6	Mean: 33.25 years (26–49 years)	Monocentric	Mean: 41 months (IR: 4–53)
Schwandner et al. [30]	*n* = 2/21	ND	Bicentric	Mean 12 months (range: 3–18)—(all the etiologies)
Cui et al. [31]	*n* = 1/9	27 years	Monocentric	12 months
Oom et al. [32]	*n* = 11/26	ND	Monocentric	Median: 14 months (range = 3–50)
Husain et al. [33]	*n* = 9/37	ND	Monocentric	4 weeks postoperatively
Bai et al. [34]	*n* = 3/21	ND	Monocentric	ND
Zimmerman et al. [35]	*n* = 9/21	ND	Monocentric	Median: 15 months (range: 4–66)
Khanduja et al. [36]	*n* = 20	Mean: 30 years (range 18–40)	Monocentric	Mean: 40 months (range: 5–75)
Veronikis et al. [37]	*n* = 22/34	ND	Monocentric	All patients were evaluated postoperatively at 6 weeks, 6 months, 1 year, and yearly thereafter
Khanduja et al. [38]	*n* = 27/48	ND	Monocentric	Mean: 16 months (range 6 weeks- 5 years)
Russell and Gallagher [39]	*n* = 6/32	ND	Monocentric	Mean > 10 years
Browning et al. [40]	*n* = 18/390	ND	Monocentric	6 months
Tong et al. [27]	*n* = 5/16	ND	Monocentric	Median: 10.2 months (range: 8–36)—All the etiologies

Articles	Procedures performed (*n* performed /* n*)	Anatomical success	Functional results (no anal incontinence)	Diverting stoma	Morbidity
Norderval et al. [26]	Autologous fat graft injection: *n* = 9/9	*n* = 8/9	ND	ND	*n* = 1 (all etiologies)
Mukwege et al. [28]	- Laparoscopic dissection rectal and vaginal suture *n* = 2/8 - Laparoscopic rectal resection *n* = 1/8 - Laparoscopic dissection "pull trought" technic *n* = 5/8	*n* = 2/2 *n* = 1/1 *n* = 4/5	*n* = 6/8	None	None
De Weerd et al. [29]	Autologous fat graft injection *n* = 4/4	*n* = 4/4	*n* = 4	n = 3/4	*n* = 2/6
Schwandner et al. [30]	Surgisis mesh: *n* = 2/2	*n* = 1/2	ND	ND	*n* = 4/21 (all the etiologies)
Cui et al. [31]	Martius flap *n* = 1	*n* = 1/1	*n* = 1	n = 1/1	Wound swollenness *n* = 3/9 (all the etiologies)
Oom et al. [32]	Puborectal sling interposition *n* = 11/11	*n* = 7/11	ND	ND	Wound infection *n* = 11/26 (all the etiologies)
Husain et al. [33]	Transperineal ELC *n* = 8/9 + Sphincteroplasty *n* = 2	*n* = 7/8	ND	n = 1/9	ND
Bai et al. [34]	Rectal ELC *n* = 2/3 Transvaginal ELC *n* = 1/3	v = 2/2 *n* = 1/1	ND	ND	ND
Zimmerman et al. [35]	ERAF *n* = 9/9	*n* = 4/9	*n* = 8/16	ND	Wound infection *n* = 2/21 dyspareunia *n* = 4/8 (all the etiologies)
Khanduja et al. [36]	ERAF + sphincteroplasty *n* = 20/20	*n* = 14/20	n = 14/20	None	ND
Veronikis et al. [37]	ERAF *n* = 22/22 + Overlapping sphincteroplasty *n* = 15/22 or perineal reconstruction with pubococygeal muscle and deep transverse perineal muscle *n* = 19/22	*n* = 20/22	*n* = 18/22	None	Mechanical disimpaction *n* = 2 urinary retention (*n* = 3) fecaloma (n = 3) wound disrupton (*n* = 2)
Khanduja et al. [38]	ERAF *n* = 27/27		*n* = 16/27	None	*n* = 6/48 *n* = 2 hématoma *n* = 2 n fecaloma *n* = 2 urinary infection
Russell and Gallagher [39]	ERAF *n* = 6/6	*n* = 5/6	*n* = 22/32 (all the etiologies)	None	none
Browning et al. [40]	Flap spliting technic* n* = 18/18	v = 18/18	*n* = 16/18	None	ND
Tong et al. [27]	Ovesco clip* n* = 5/5	*n* = 1/5	*n* = 4/5	ND	ND

*ERAF* endorectal advancement flap, *ELC* excision and layered closure, *IQR* interquartile range

The number of patients included in each study varied from 1 to 60. Only six studies focused on post-OASI RVF [6, 17, 18, 22, 24, 36]. Finally, 595 women and/or procedures were considered in this review. The mean or median ages were available in 12 studies and were between 18 and 66 years. Only two studies reported body mass index (BMI) [5, 6]. Venara et al. [6] reported a mean BMI of 27 kg/m² (± 5.5), while Karp et al. [5] reported that 26.4% of the patients had a BMI ≥ 30 kg/m².

Among the 595 patients included, the most performed procedure was the ERAF (*n* = 180) followed by the Musset procedure (*n* = 65) and transperineal ELC (*n* = 149). The success rate was reported between 37.5% and 100% depending on the procedure (Table 3).

**Table 3:** Synthesis of the number of procedures performed according to the prospective and retrospective characteristic of the studies and their anatomical results

Procedure	Number of studies	Number of procedures	Anatomical success
Endorectal avandcement flap	*n* = 11 (Retro: *n* = 6/Prosp: n = 5)	*n* = 180 (Retro: *n* = 96/Prosp: *n* = 84)	*n* = 98/180 (Retro: *n* = 39; 20–100%/Prosp: *n* = 59; 44–91%)
Musset procedure	*n* = 14 (Retro: *n* = 12/Prosp: *n* = 3)	*n* = 243 (Retro: *n* = 206/Prosp: *n* = 37)	*n* = 184/243 (Retro: *n* = 152; 65–100%/Prosp: *n* = 7; 64–100%)
Transperineal ELC			
Transvaginal ELC	*n* = 4 (Retro: *n* = 3/Prosp: *n* = 1)	*n* = 62 (Retro: *n* = 61/Prosp: *n* = 1)	*n* = 60/62 (Retro: *n *= 59; 86–100%/Prosp: *n* = 1; 100%)
Rectal ELC	*n* = 1 (Prosp)	*n* = 2	*n* = 2/2 (100%)
Simple suture	*n* = 1 (Retro)	*n* = 1	*n* = 1/1 (100%)
Martius flap	*n* = 5 (Retro: *n* = 4/Prosp: *n* = 1)	*n* = 21 (Retro: *n* = 20/Prosp: *n* = 1)	*n* = 13/21 (Retro: *n* = 12; 50%–100%/Prosp: *n* = 1; 100%)
Gracilis flap	*n* = 3 (Retro exclusively)	*n* = 14	*n* = 10/14 (66–100%)
Glue	*n* = 1 (Retro)	*n* = 10	*n* = 4/10 (40%)
Plug	*n* = 1 (Retro)	*n* = 7	*n* = 3/7 (42%)
Puborectal sling interposition			
Flap splitting technique			
Vaginal flap	*n* = 2 (Retro)	*n* = 9	*n* = 4/9 (37.5–100%)
Autologous fat graft	*n* = 2 (Retro: *n* = 0/Prosp: *n* = 2)	*n* = 13 (Prosp: *n* = 13)	*n* = 12/13 (92.4%)
Ovesco clip	*n* = 2 (Retro: *n* = 1; Prosp: *n* = 1)	*n* = 14 (Retro: *n* = 9/Prosp: *n* = 5)	*n* = 4/14 (Retro: *n* = 3/9; 33%/Prosp: *n* = 1/5; 20%)
Lift	*n* = 1 (Retro)	*n* = 1	*n* = 1/1 (100%)
Plug	*n* = 1 (Prosp)	*n* = 2	*n* = 1/2 (50%)
Abdominal access			
Rectal and vaginal suture	*n* = 1 (Prosp)	*n* = 2	*n* = 2/2 (100%)
Rectal resection	*n* = 1 (Prosp)	*n* = 1	*n* = 1/1 (100%)
Pull-through technique	*n* = 1 (Prosp)	*n* = 5	*n* = 4/5 (80%)
Spontaneous healing	*n* = 3 (Retro)	*n* = 8	ND
Total		*n* = 595	

*Prosp* prospective study, *Retro* retrospective study, *ELC* excision and layered closure, *ND* not defined

Stoma construction was reported in 24 studies, but only 10 reported the number of stoma constructions in the obstetric RVF group [6, 14–16, 22, 23, 25, 29, 31, 33]. In these 10 studies, 66 stomas were performed in 132 women. The significance of the stoma in improving the surgical success rate was only assessed in the study of Venara et al. [6], which reported that a stoma does not significantly improve the success rate (OR = 1.46; 95% CI: 0.31–6.91).

The functional results and the postoperative outcomes were not extensively reported in the literature (Tables 1 and 2). Furthermore, when morbidity was reported, the authors did not specify the etiology of the RVF.

### Results of the different procedures

The endorectal advancement flap (ERAF) is the most studied procedure in the literature. Six retrospective studies reported heterogeneous results ranging between 20 and 100%, while 5 prospective studies reported results from 44 to 91% (Table 3).

Excision and layered closure (ELC) was the second most frequently studied procedure. Three surgical accesses can be used: (1) the transperineal access (8 retrospective studies and 1 prospective study) reporting results from 80.5 and 100% and 87.5%, respectively; (2) the transvaginal access (3 retrospective studies and 1 prospective) reporting results from 83.3 to 100% (Table 3).

### Meta-analysis

**Fig. 2:**
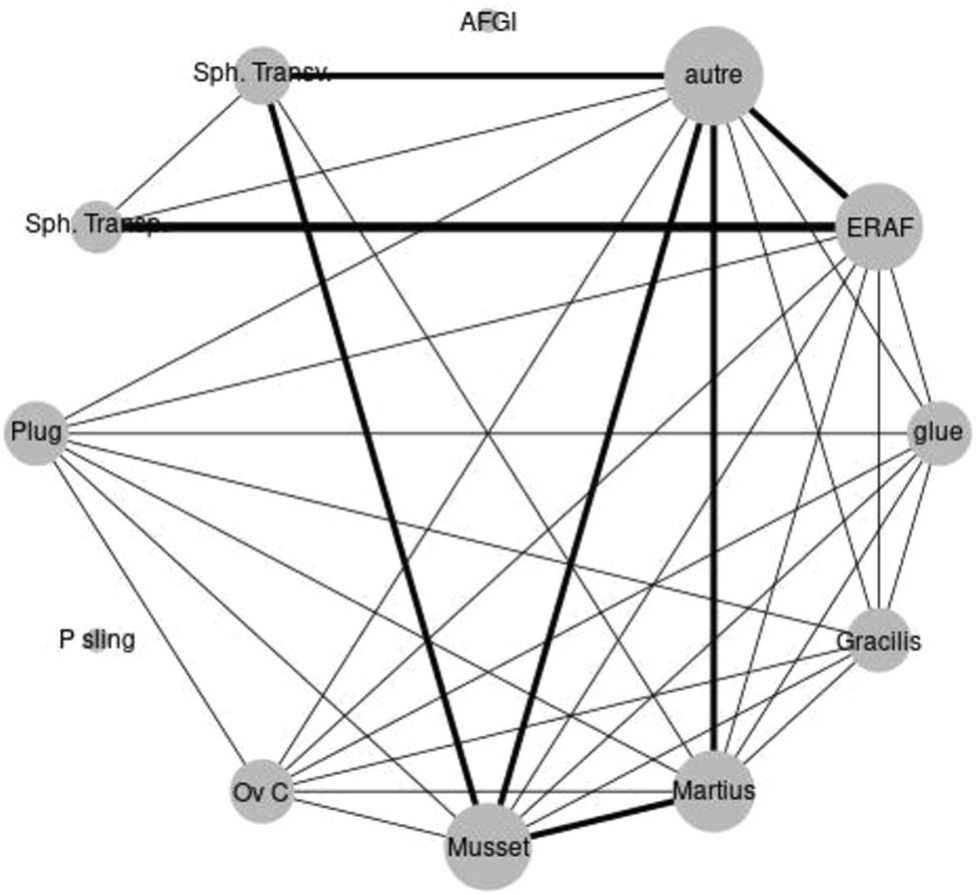
Pairwise comparisons of the anatomical results presented in the considered studies (each line corresponding to a direct pairwise comparison, the width of the line illustrating the number of studies presenting such a pairwise comparison). *ERAF* endorectal advancement flap, *Ov C* Ovesco clip, *Sph transp* transperineal sphincteroplasty (excision and layered closure), *sph transv* transvaginal sphincteroplasty (excision and layered closure), *P Sling* puborectal sling

**Fig. 3:**
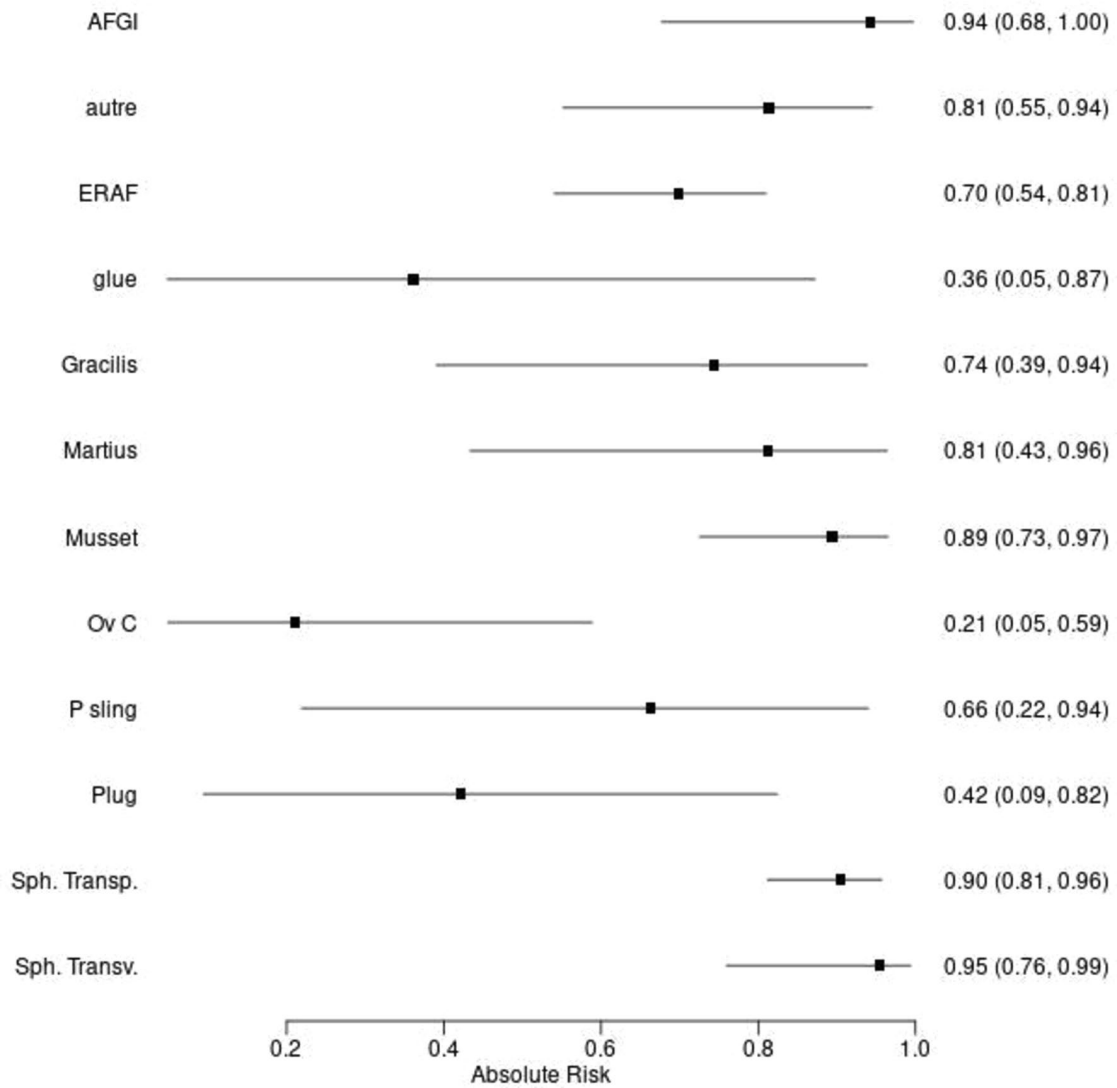
Absolute proportion of anatomical corrections of the defect depending on the considered treatment, associated with the 95% confidence interval. *ERAF* endorectal advancement flap, *Ov C* Ovesco clip, *Sph transp* transperineal sphincteroplasty (excision and layered closure), *sph transv* transvaginal sphincteroplasty (excision and layered closure), *P Sling* puborectal sling

**Fig. 4:**
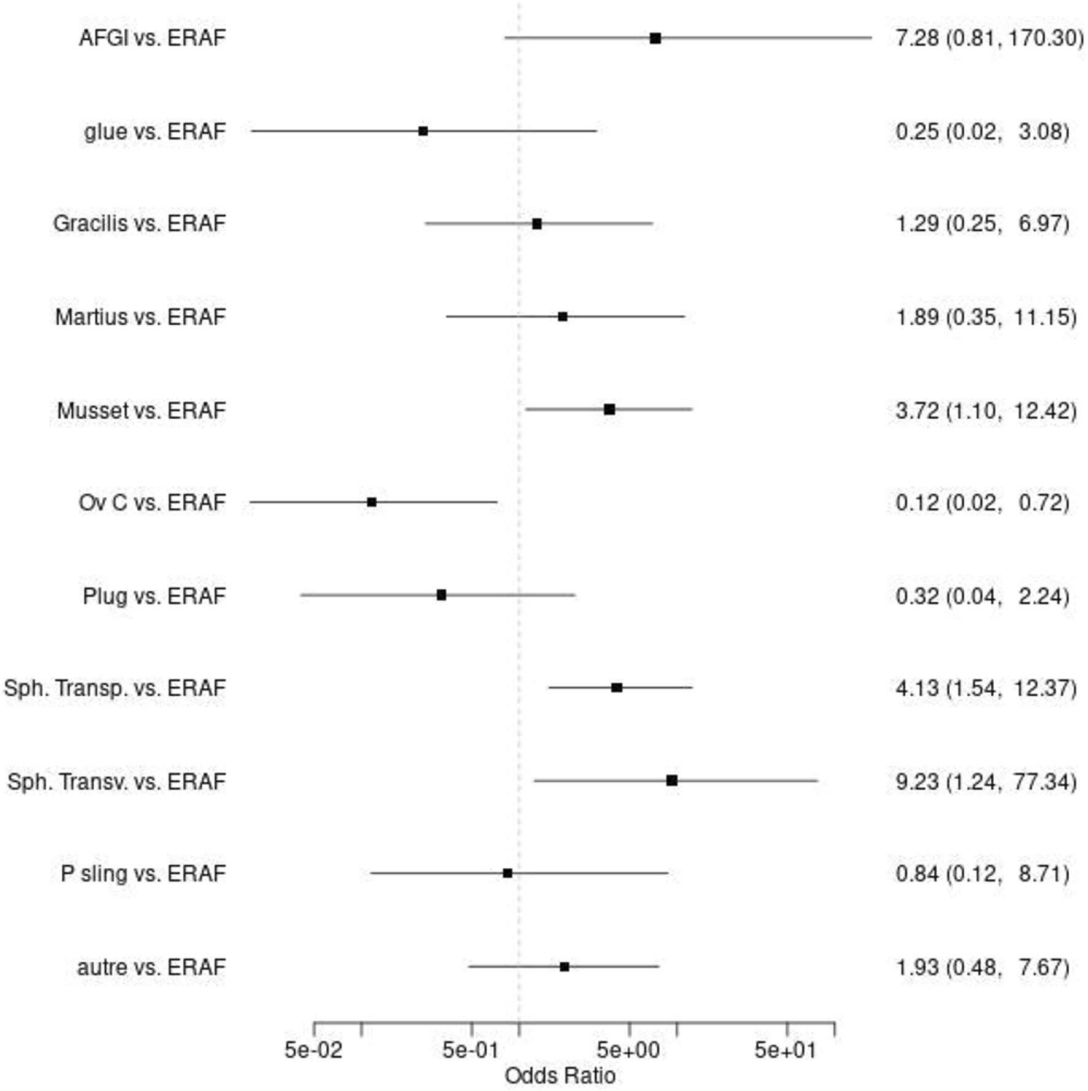
Comparison of the odds ratios for each of the procedures studied (reference: endorectal advancement flap). *ERAF* endorectal advancement flap, *Ov C* Ovesco clip, *Sph transp* transperineal sphincteroplasty (excision and layered closure), *sph transv* transvaginal sphincteroplasty (excision and layered closure), *P Sling* puborectal sling

Absence of postoperative fecal incontinence and construction of a diverting stoma were not mentioned frequently enough to allow performing a meta-analysis considering these endpoints. The only possible endpoint considered was the anatomical correction of the defect. A list of direct pairs for comparison presented by the considered studies is given in Fig. 2 (each line corresponding to a direct pair for comparison, the width of the line illustrating the number of studies presenting such a pair for comparison). The absolute proportions of anatomical corrections of the defect depending on the considered treatments, associated with their 95% confidence intervals, are presented in Fig. 3. The Musset procedure, transvaginal ELC and transperineal ELC all presented significantly better results than the ERAF procedure (OR of 4.29; 95% CI: 1.18–16.14 for Musset procedure, 11.84; 95% CI: 2.18–91.80 for transvaginal ELC and 3.56; 95% CI: 1.26–10 for transperineal ELC). The odds ratios for each of the procedures studied are presented in Fig. 4.

### Quality of the studies included

The quality of the studies included was low because of the lack of randomized controlled studies. However, most of the studies had a good design and analysis (Table 4).

**Table 4:** JBI checklist for cohort and case studies of those included

	JBI checklist = cohort study											JBI checklist = case series									
	Were the two groups similar and recruited from the same population?	Were the exposures measured similarly to assign people to both exposed and unexposed groups?	Was the exposure measured in a valid and reliable way?	Were confounding factors identified?	Were strategies to deal with confounding factors stated?	Were the groups/participants free of the outcome at the start of the study (or at the moment of exposure)?	Were the outcomes measured in a valid and reliable way?	Was the follow-up time reported and sufficient to be long enough for outcomes to occur?	Was follow-up complete, and if not, were the reasons for loss to follow-up described and explored?	Were strategies to address incomplete follow-up utilized?	Was appropriate statistical analysis used?	Were there clear criteria for inclusion in the case series?	Was the condition measured in a standard reliable way for all participants included in the case series?	Were valid methods used for identification of the condition for all participants included in the case series?	Did the case series have consecutive inclusion of participants?	Did the case series have complete inclusion of participants?	Was there clear reporting of the demographics of the participants in the study?	Was there clear reporting of clinical information of the participants?	Were the outcomes or follow-up results of cases clearly reported?	Was there clear reporting of the presenting site(s)/clinic(s) demographic information?	Was statistical analysis appropriate?
Venara et al. [6]	Yes	Yes	Yes	Yes	Yes	Yes	Yes	Yes	Yes	No	Yes										
Beksac et al. [13]												No	Yes	Unclear	No	No	Yes	Yes	Yes	Yes	Yes
Norderval. et al. [26]												Yes	Yes	Yes	Yes	Unclear	Yes	Yes	Yes	Yes	Yes
Reisenauer et al. [14]												Yes	Yes	Unclear	Yes	Yes	Yes	Yes	Yes	Yes	Yes
Hull et al. [9]	Yes	Yes	Yes	Yes	Yes	Yes	Yes	Yes	Yes	Yes	Yes										
Songne et al. [15]	No	Yes	Yes	No	No	Yes	Yes	Yes	Yes	NA	Yes										
Devesa et al. [16]	No	Yes	Yes	No	No	Yes	Yes	Yes	Yes	NA	Yes										
Chew et al. [17]	Yes	Yes	Yes	No	No	Yes	Yes	Yes	No	No	Yes										
Rahman et al. [18]	Yes	Yes	Yes	No	No	Yes	Yes	No	Yes	NA	Yes										
Sonoda et al. [8]	No	Unclear	Yes	Yes	Yes	Yes	Yes	No	Yes	NA	Yes										
Soriano et al. [19]	No	Yes	Yes	Yes	Yes	Yes	Yes	Yes	Yes	NA	Yes										
Ozuner et al. [20]	No	Yes	Yes	Yes	Yes	Yes	Yes	Yes	Yes	NA	Yes										
MacRae et al. [21]	No	Yes	Yes	Yes	Yes	Yes	Yes	No	No	Yes	Yes										
Reisenauer et al. [22]	Yes	Yes	Yes	No	No	Yes	Yes	Unclear	Unclear	Unclear	Unclear										
Karp et al. [5]	Yes	Yes	Yes	Yes	Yes	Yes	Yes	No	Yes	NA	Yes										
Zmora et al. [23]												Yes	Yes	Yes	Yes	Yes	Yes	Yes	Yes	Yes	Yes
Frontali et al. [25]	Yes	Unclear	Yes	Yes	Yes	Yes	Yes	Yes	Yes	NA	Yes										
Corman et al. [24]	Yes	Yes	Yes	No	No	Yes	Yes	No	Unclear	Unclear	NA										
Mukwege et al. [28]												Yes	Yes	Yes	Yes	Yes	Yes	Yes	Yes	Yes	Yes
De Weerd et al. [28]												Unclear	Unclear	Yes	No	Yes	No	Yes	Yes	No	Yes
Schwandner et al. [30]	No	Yes	Yes	Yes	No	Yes	Yes	No	Yes	NA	Unclear										
Cui et al. [31]	No	Yes	Yes	Yes	No	Yes	Yes	No	Yes	NA	Yes										
Oom et al. [32]	No	Yes	Yes	Yes	No	Yes	Yes	Yes	No	No	Yes										
Husain et al. [33]	Yes	Yes	Yes	Yes	No	Yes	Yes	No	Yes	NA	Yes										
Bai et al. [34]	No	Unclear	Unclear	Yes	No	Yes	Unclear	Unclear	Unclear	Unclear	No										
Zimmerman et al. [35]	No	Yes	Yes	Yes	Yes	Yes	Yes	No	Yes	NA	Yes										
Khanduja et al. [36]	Yes	Yes	Yes	Yes	No	Yes	Yes	Yes	Yes	NA	Yes										
Veronikis et al. [37]	Yes	Yes	Yes	Yes	Yes	Yes	Yes	No	Yes	NA	Yes										
Khanduja et al. [36]	Yes	Yes	Yes	No	No	Yes	Yes	Yes	Yes	NA	Yes										
Russell et al. [39]	Unclear	Yes	Yes	No	No	Yes	No	Yes	Yes	NA	Yes										
Browning et al. [40]	No	Yes	Yes	No	No	Yes	Yes	No	No	No	Yes										
Tong et al. [27]	Unclear	Unclear	Yes	Yes	Yes	Yes	Yes	No	Yes	NA	Yes										

## Discussion

### Main findings

In this systematic review and meta-analysis including 18 retrospective studies and 14 observational prospective studies, 595 women undergoing surgery for obstetric RVF were considered. The quality of the studies included was low because of the lack of prospective randomized studies. Nineteen procedure types were described and assessed. Most of the patients (*n* = 180) underwent ERAF followed by ELC (*n* = 213) and the Musset procedure (*n* = 65). A diverting stoma was performed in 66 of the 132 women in which it was reported. Only 13 studies reported the functional results of the procedure. In the meta-analysis, the ELC and Musset procedure gave the best results.

### Strength

To our knowledge, this is the first systematic review including only obstetric RVF women. The meta-analysis advocates proposing new principles and standards for RVF closure. The arm-based network meta-analysis based on the Zhang et al. Bayesian hierarchical models [12] is a relatively innovative methodology, particularly adapted to the context of the studies analyzed. Classical meta-analysis, focusing only on directly paired comparisons between two specific treatments, could not be considered because of the many possible surgeries [41]. Network meta-analysis allows considering a large number of possible treatments in a single analysis, even if the collection of studies identified through the systematic review does not specifically report each of the possible treatment pairs for comparison [42]. Classical contrast-based network meta-analysis methods do not allow considering single-arm studies (i.e., focusing the study on a given surgery) but only studies reporting results as relative effects (e.g., odds ratios). Only arm-based network meta-analysis methods could allow evaluation of both relative treatment effects (e.g., odds ratios) and absolute risks (e.g., overall percentage of success for each arm) while considering both single arm studies and studies comparing several treatments.

### Limitations

None of the studies were randomized controlled studies, and the quality assessment of the studies showed some to be inferior. However, to improve the quality of the data collected, the case reports were excluded considering that they only related to focal procedures. The patients included in the present meta-analysis came from cohort studies including all consecutive patients with RVF, limiting the selection bias, even if only one patient was included in the study. The perioperative management of the patients might also represent a bias in the analysis. Indeed, there is a lack of information about constructing a diverting stoma, and most of the studies do not report the results according to such a construction. To date, the role of diverting stoma construction is debated as some studies conclude it does not improve the success rate [6, 43] while others counter this [44]. This difference may be due to the surgical procedure performed or to the etiology of the RVF included, obstetrical RVF having a higher rate of success than the other etiologies [5, 45]. As information about the stoma is not detailed in the studies, no assessment was feasible in a meta-analysis. Also, the predictors of the success of the surgery, such as a higher BMI and the number of repairs, were not considered in the present analysis [45, 46]. Finally, the description of the RVF was poorly reported in the studies and was therefore not collected. This may represent a bias. However, only obstetric RVFs were included in the present meta-analysis. This limits the risk of bias because obstetric RVFs are usually short and associated with sphincter defects.

### Interpretation

While obstetric RVFs have a good prognosis with a reported healing rate between 40 and 100% [6, 8, 13, 15], women need a mean of 2.5 (± 1.7) procedures to heal the fistula [47]. The ASCRS recommends favoring the sphincter-sparing procedure [48], explaining why most of procedures performed are ERAFs despite their variable results from 50 to 70% [48]. For RVF, the German guidelines state that no specific procedure can be recommended according to the literature [48]. At first sight, the ERAF may represent an easy procedure with low morbidity, but a recent meta-analysis reports morbidity in 53.6% of the ERAFs performed in ano-perineal fistula [49]. The main complications reported were flap disruption and fecal incontinence [49]. In the context of RVF, a flap disruption can lead to an increase of the RVF opening, leading to consideration of the diverting stoma to increase the patient's quality of life or to reduce infection [50]. The present meta-analysis suggests that the Musset procedure or excision and layered closure could yield the best results. Literature on the Musset procedure is scarce and does not provide information on the technique's morbidity [20] as comprehensively as for ELC. Due to this lack of information in the literature, it is not possible to make a recommendation, but the common aim of the Musset procedure and ELC is to repair the sphincter, indicating that sphincteroplasty should be performed during RVF repair.

Finding the best way to repair obstetric fistulas is an issue of utmost significance as it affects individuals very personally as well as communities more broadly, especially in LMIC [51], and it reduces quality of life [45]. Furthermore, as the number of the procedures is a risk factor for failure of further surgeries [45], the most efficient procedure has to be determined. Further randomized controlled study is required to confirm whether sphincteroplasty provides better anatomical and functional results than ERAF.

## Conclusion

Obstetric RVF is mainly repaired using ERAF, with poor results. ELC and the Musset procedure, which both allow sphincteroplasty, had better anatomical results. The literature review did not allow conclusions to be drawn about the value of diverting stoma in that context. Randomized controlled trials in the literature are required to assess and compare the anatomical and functional results of ERAF and sphincteroplasty as well as their related morbidity rates.

## Supplementary Information

Below is the link to the electronic supplementary material.

Supplementary file1 (PDF 678 KB)

## Data Availability

No datasets were generated or analyzed during the current study.
