# Correction: Sphincter repair procedures may be favored in the treatment of obstetrical recto‑vaginal fistula: a systematic review of the literature and meta‑analysis

**DOI:** 10.1007/s10151-025-03168-6

**Published:** 2025-05-07

**Authors:** A. Venara, E. Houlet, E. Poupard, M. André, P. E. Bouet, J. Gillet, J. F. Hamel

**Affiliations:** 1 Faculty of Health, Department of Medicine, University of Angers, Angers, France; 2 Department of Digestive Surgery, University Hospital of Angers, 4 rue Larrey, Angers Cedex 9, 49933 Angers, France; 3 IHFIH, UPRES, University of Angers, 3859 Angers, EA France; 4 The Enteric Nervous System in Gut and Brain Disorders, Université de Nantes, INSERM, TENS, IMAD, 44000 Nantes, France; 5 Department of Gynecology and Obstetrics, University Hospital of Angers, 4 rue Larrey, angers cedex 9, 49933 Angers, France; 6 Department of Biostatistics, La Maison de La Recherche. University Hospital of Angers, 4 rue Larrey, Angers Cedex 9, 49933 Angers, France; 7 Department of Visceral Surgery, CHU Angers, 4 rue Larrey, 49933 Angers Cedex 09, 49933 Angers, France

**Correction: Techniques in Coloproctology** 10.1007/s10151-025-03133-3

In this article, the author’s name E. Houlet was incorrectly written as H. Houlet. The original article has been corrected.

